# Effects of ecological restoration projects on changes in land cover: A case study on the Loess Plateau in China

**DOI:** 10.1038/srep44496

**Published:** 2017-03-21

**Authors:** Jun Zhao, Yanzheng Yang, Qingxia Zhao, Zhong Zhao

**Affiliations:** 1 College of Forestry, Northwest A&F University, Yangling, Shaanxi 712100, P. R. China; 2 Key Comprehensive Laboratory of Forestry, Shaanxi Province, P. R. China; 3 Key Laboratory of Silviculture on the Loess Plateau, State Forestry Administration, Shaanxi Province, P. R. China; 4 Ministry of Education Key Laboratory for Earth System Modeling, Department of Earth System Science, Tsinghua University, Beijing, China.

## Abstract

Changes in land cover have become key components of global environmental change and represent the impact of human activity. To better understand the fundamental processes of land transition characteristics before and after the implementation of ecological programmes, we determined the dominant systematic changes in land cover in Yongshou, a hilly-gully region on the Loess Plateau. This was achieved by performing an in-depth analysis of a cross-tabulation matrix and a modified spatial dynamic degree model. Our results indicated that (1) forest land and cultivated land were the most important land cover types in Yongshou and their persistence would greatly affect the landscape pattern of the entire region; (2) the most significant changing signals in the study area during the periods 1992–2000 and 2000–2013 were from immature forest land to forest land, cultivated land to orchards and orchards to construction land; and (3) the region that experienced the most changes during 1992–2000 was the densely populated county seat of Yongshou; however, from 2000–2013, the region of most changes was Changning, a town located in the northcentral region of Yongshou. These findings reveal the main characteristics of the land cover changes in this region and provide insight into the processes underlying these changes.

Changes in land cover have been a key research priority and a local environmental issue. They are becoming a primary determinant of global change^1^,^2^, having major effects on biodiversity^3^, hydrology^4^, global biogeochemical cycling mechanisms^5^, climate change^6^ and ecosystem services^7^. A better understanding of the mechanisms underlying changes in land cover is of increasing interest in global change and environmental research^8^,^9^. Land cover patterns are caused by human activity and natural factors, and the influence of human factors on the dynamics of land cover changes has become increasingly obvious^10^. Therefore, identifying the driving forces and the specific impacts on the structure and dynamics of land cover from a historical perspective is important and necessary.

As a result of the rapid social changes and population growth in China under the planned economy from 1953 to 1978 and the subsequent economic reforms of 1978, a series of ecological problems has emerged. For example, deforestation for the expansion of subsistence crop production has apparently induced high rates of water and soil erosion, biodiversity loss and land degradation on the Loess Plateau in China^11^,^12^,^13^,^14^. These severe ecological problems greatly affect the quality of life and survival of local populations^15^. These problems have promoted the Chinese government to seek more effective mitigation strategies, such as the “Three North” Shelterbelt Development Program (TNSDP) that was implemented in 1978. As an important initiative promoting ecological restoration to combat ecological degradation, the TNSDP helped improve ecological conditions in ecologically vulnerable regions^16^. Degradation in an area of the Loess Plateau with 40% soil and water erosion has been mitigated by the first phase of the programme, which was completed in 2000^17^. Land cover changes, low forest cover, soil erosion and ecological environmental deterioration continue to be closely monitored. Subsequently, the second phase of the TNSDP (2000), the Grain for Green Project (GFGP, implemented in 1999) and the Natural Forest Protection Project (NFPP, implemented in 2000) have been vigorously implemented by government.

Considerable research progress has been made in mapping land cover, monitoring the dynamics and driving forces, and identifying regional environmental benefits^18^,^19^. Many studies have highlighted that land cover change is a widespread phenomenon on the Loess Plateau in China^20^,^21^,^22^,^23^,^24^. These studies have mostly focused on land cover changes and their influences on the Loess Plateau over different periods. Change in vegetation has been a major concern since the implementation of ecological programmes. The restoration of vegetation and anthropogenic changes have greatly reduced sediment transport in the Yellow River^25^,^26^. In addition, there have been large changes in the soil carbon and nitrogen pools following the implementation of ecological programmes^27^,^28^. Furthermore, land cover change has had a large influence on the regional climate and plant phenology^29^,^30^. To fully understand the influence of land cover change, the current status and trends of land cover change must be identified.

Among the various approaches for detecting land cover change, the Markov model, which is a quantitative analysis method proposed by Andrey Markov^31^, and the dynamic degree model have been widely used by researchers^32^. Most studies of land cover change have focused primarily on analysing changes in land cover, detecting rate changes and evaluating the amplitude of different land cover types and large inter-category transitions^33^,^34^. However, such studies do not consider the detailed transition processes, and their interpretations of the transition matrix might, therefore, fail to reveal systematic processes^35^. For example, a study might focus on the largest transition of change; however, small changes can have a major influence on the environment. An in-depth analysis based on transition matrices can reveal swap, gross gains, gross losses and net changes to ascertain whether land cover transitions are systematic or random^36^. Distinguishing between systematic and random changes allows researchers to consider not only the transitions but also the quantity and spatial distribution of land cover types. The dynamic degree model of changes in land cover that was proposed by Liu^32^ can be used to measure and compare the activity of land cover change rapidly and to accurately describe its intensity. This model has been widely used in many administrative areas^37^,^38^,^39^,^40^, and it provides an effective way to measure the comprehensive dynamic degree of changes in land cover. However, the traditional dynamic degree model can only yield the dynamics of an entire area, and internal spatial dynamics are difficult to detect using this method. Therefore, it considers only one-way transition processes of land cover change, and details regarding gains are not considered. As a result, regions with low transitions and rapid gains of the characteristics of interest, such as urban areas, are massively underestimated.

To better understand the influences of ecological programmes on land cover change, it is necessary not only to detect the quantity and direction of land cover change but also to obtain accurate information about the potential processes underlying this change. In this study, we conducted an in-depth statistical assessment by evaluating a transition matrix and a modified land cover change dynamic model at the village level. The major objectives of this study were to (1) quantify the changes in land cover processes and trends, (2) analyse the systematic and random transitions and (3) detect the spatial dynamics and driving forces of changes in land cover.

## Results

### Changes in land cover change processes and trends

**Figure 1:**
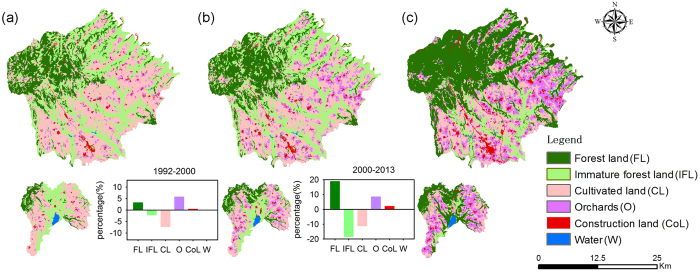
Area and spatial distribution of the land cover types in the study area in 1992 (a), 2000 (b) and 2013 (c). The two bar plots of the land cover changes during the periods of 1992–2000 and 2000–2013 represent the changes in the information during the two periods. FL, forest land; IFL, immature forest land; CL, cultivated land; O, orchards; CoL, construction land; W, water. The maps were generated with ArcGIS 10.2: http://www.esri.com/.

The results obtained from multi-temporal land cover analyses reveal extensive changes in land cover and land cover trends in the study area (Table 1, Table S1, Fig. 1). The study area was classified into six land cover categories, including forest land, immature forest land, cultivated land, orchards, construction land, and water (Table S3, Fig. 1). The transitions between land cover types showed remarkable differences between periods; for example, the transition from cultivated land to orchards was the most common transition during 1992–2000. The implementation of ecological policies in 2000 shifted the predominant land cover transition to the conversion of immature forest land to forest land in 2000–2013 (Fig. 1).

We observed that the predominant land cover type changed from cultivated land to forest land during 1992–2000 (Table 1). In 1992, cultivated land occupied the largest area (42.39%), followed by immature forest land (33.66%), forest land (17.93%), and other land cover types (6.02%). The predominance of cultivated land (35.06%) continued in 2000, although the relative percentage was lower than before, and the relative percentage of orchards (9.51%) had increased. The relative percentages of immature forest land (31.46%) and forest land (21.23%) remained high, and the percentage of construction land (2.41%) showed an upward trend. In 2013, the predominant land cover type became forest land (40.17%), which mainly transitioned from immature forest land (17.71%) and a small amount of cultivated land (1.58%). Orchard area (18.10%) rapidly increased because of the transition from cultivated land. Town expansion caused a continuous increase in construction land (4.61%) as well.

The study area experienced different transition tendencies during the two periods. From 1992–2000, forest land, orchards and construction land showed increasing trends, whereas the areas of cultivated land, immature forest land and water showed decreasing trends. The highest gain was observed in orchards (6.13%), followed by forest land (3.38%) these gains mainly represented conversion from immature forest land (3.11%). The highest loss occurred in cultivated land (7.77%) which were mainly converted to orchards, followed by immature forest land (3.60%) and these losses were mainly converted to forest land. Cultivated land experienced the greatest total changes (8.21%) and the net change was the highest (7.33%), whereas its swap change was only 0.88%. The results indicated that cultivated land predominantly experienced changes in quantity rather than in swap. Similar to cultivated land, forest land, orchards and construction land mostly experienced quantity changes, whereas immature forest land and water predominantly experienced both quantity changes and swap. In 2000–2013, forest land experienced the highest gain (19.34%), lower gains were observed for orchards (9.04%) and construction land (2.21%). Losses in immature forest land were the highest due to the implementation of reforestation policy (18.84%), followed by cultivated land (11.74%). The highest total changes occurred in forest land (19.73%), followed by immature forest land (19.27%). The total changes in cultivated land, orchards, construction land and water were 12.25%, 9.51%, 2.24% and 0.10% respectively. All of the land cover transitions except those to water were dominated by changes in quantity. Moreover, the transition from immature forest land to forest land was unexpectedly larger than the persistence of immature forest land from 2000–2013 (Table 1).

**Figure 2:**
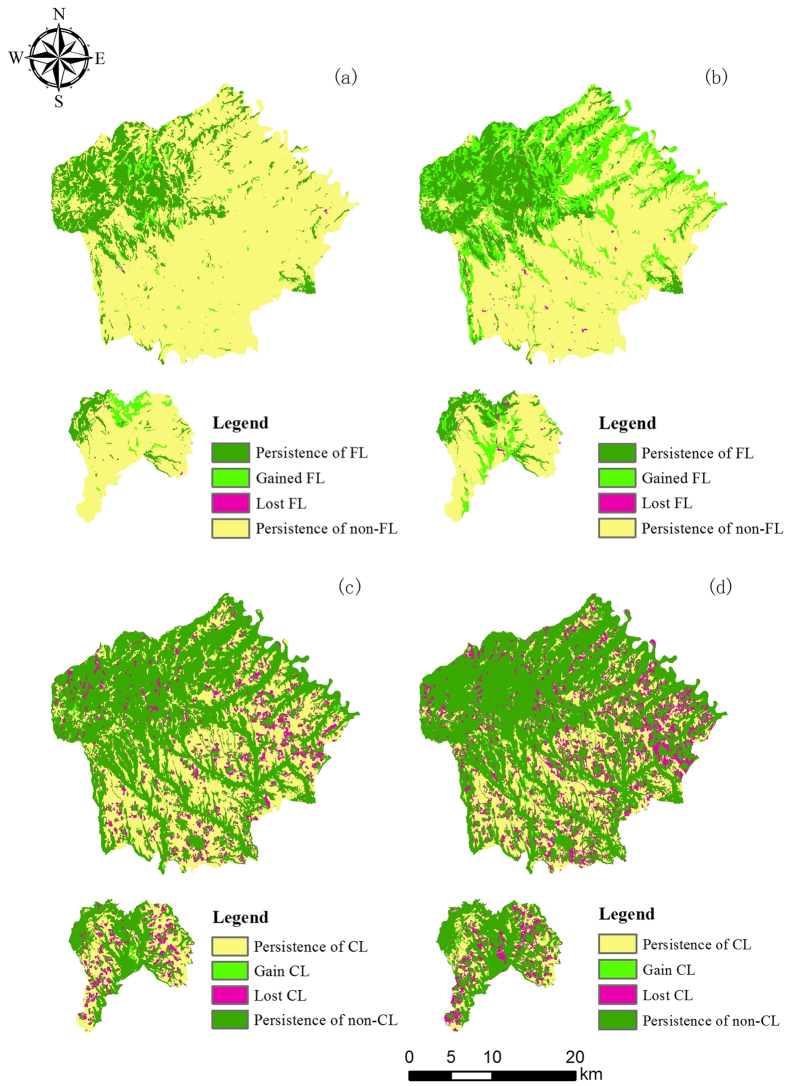
Spatial representation of the gains, losses, and persistence experienced by (a) FL (1992–2000), (b) FL (2000–2013), (c) CL (1992–2000) and (d) CL (2000–2013). The maps were generated with ArcGIS 10.2: http://www.esri.com/.

Forest land and cultivated land are the most important land cover types in Yongshou, and their persistence greatly impacts the landscape pattern of the entire area (Fig. 2). The persistence of forest land was 17.85% from 1992–2000 (Fig. 2a) and increased to 20.80% from 2000–2013 (Fig. 2b). The gain of forest land was 3.38% from 1992–2000 (Fig. 2a) and increased to 19.34% from 2000–2013 (Fig. 2b), representing a significant gain trend. Figure 2c and d show that the persistence of cultivated land was 34.62% from 1992–2000 and decreased to 23.25% from 2000–2013. The loss of cultivated land increased from 7.77% (Fig. 2c) to 11.74% (Fig. 2d), suggesting a tendency for loss rather than persistence or gain.

### Detection of spatial systematic and random transitions

From 1992–2000, the difference and the combined relative difference between the observed and the expected gains under a random process of change (D_ij_ and R_ij_, respectively) for the transition between cultivated land and orchards were 3.38% and 1.25%, respectively (Table 2). Thus, the 6% transition of land cover types from cultivated land to orchards was caused by systematic change. Specifically, when orchards increased, they replaced cultivated land at a predictable rate, and new orchards tended to systematically arise from cultivated land. The D_ij_ and R_ij_ between the observed and the expected gains for a random change process for immature forest land to forest land were 1.72% and 1.24%, respectively, which indicated a systematic change from immature forest land and a rate different than the expected value. Because of the small amount of construction land, the D_ij_ between the observed and expected gains under a random process was 0.28%, although the R_ij_ reached 14.00% (Table 2), indicating a strong tendency for a transition from orchards to construction land. The D_ij_ and R_ij_ between the observed and expected gains for immature forest land to orchards were large and negative (−2.13% and −1.00%, respectively), implying that new orchards did not systematically arise from immature forest land. Similarly, forest land did not systematically arise from cultivated land (−1.52% and −0.87%). The D_ij_ between the observed and the expected losses under a random change process for cultivated land to orchards, immature forest land to forest land and orchards to construction land transitions were 4.94%, 1.99% and 0.29%, respectively. Thus, cultivated land was systematically lost to orchards, immature forest land was lost to forest land, and orchards were lost to construction land. The R_ij_ between orchards and construction land was 29.00%, indicating a highly significant tendency for the transition from orchards to construction land. The D_ij_ and R_ij_ values between the observed and expected losses for cultivated land to immature forest land were large and negative (−2.47% and −0.66%, respectively), implying that the loss of cultivated land to immature forest land was systematically prevented. Similarly, the loss of cultivated land to forest land was systematically prevented (−2.31%; −0.91%).

From 2000–2013, the D_ij_ and R_ij_ between the observed and the expected gains for a random change process for an immature forest land to forest land transition were 9.99% and 1.29%, respectively. Thus, a transition of 18% of the land cover from immature forest land to forest land was caused by systematic change, implying that when forest land increased, the new forest land tended to systematically arise from immature forest land. The D_ij_ and R_ij_ between the observed and the expected gains for a random change process for a cultivated land-orchards transition were 5.45% and 1.56%, respectively, indicating the systematic transition from cultivated land to orchards. These results indicate that orchards systematically arose from cultivated land at a rate greater than expected. The D_ij_ and R_ij_ between the observed and expected gains for the cultivated land to forest land transition were large and negative (−7.03% and −0.82%, respectively), implying that increases in forest land did not systematically arise from cultivated land. Similarly, orchards did not systematically arise from immature forest land (−3.07%; −0.98%), forest land did not systematically arise from orchards (−2.33%; −1.00%), and orchards did not systematically arise from forest land (−2.10%; −0.99%). The D_ij_ between the observed and the expected losses under a random change process for immature forest land to forest land, cultivated land to orchards and orchards to construction land transitions were 9.01%, 6.16% and 0.40%, respectively. Thus, immature forest land was systematically lost to forest land, cultivated land was lost to orchards, and orchards were lost to construction land. The R_ij_ values between cultivated land and orchards, orchards and construction land were 2.21% and 13.33% respectively, indicating a highly significant tendency for the transition from cultivated land to orchards and orchards to construction land. The D_ij_ values between the observed and expected losses for the cultivated land to forest land, immature forest land to cultivated land and immature forest land to orchards transitions were large and negative (−4.61%, −4.79% and −3.85%, respectively), thus implying that forest land did not systematically arise from cultivated land, cultivated land did not systematically arise from immature forest land, and that orchards did not systematically arise from immature forest land. Similarly, a negative D_ij_ for cultivated land to immature forest land (−1.59%) implied that cultivated land did not systematically convert from immature forest land.

**Figure 3:**
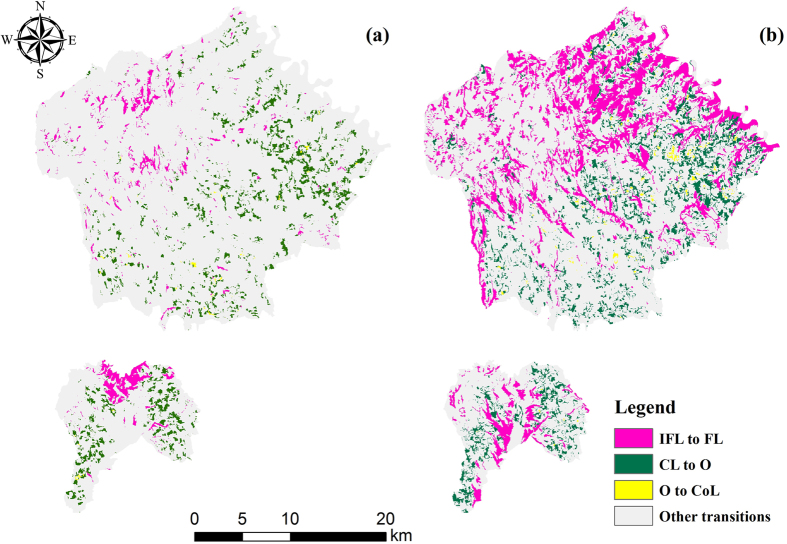
Systematic land cover transitions from1992–2000 (a) and 2000–2013 (b). The maps were generated with ArcGIS 10.2: http://www.esri.com/.

Based on the above analyses, the most dominant signals of change in the two periods comprised the following: (1) conversion from immature forest land to forest land, (2) conversion from cultivated land to orchards, and (3) conversion from orchards to construction land (Fig. 3). Although the most dominant signals of land cover change exhibited similar trends between the two periods, many differences were detected. For 1992–2000, only 3% of conversion was from immature forest land to forest land, but this value increased to 18% for 2000–2013. The continual implementation of ecological restoration programmes yielded a significant success in Yongshou. To promote economic development, the government encouraged farmers to build orchards. Therefore, the conversion from cultivated land to orchards increased continually from 6% to 9% during the two periods. In both periods, the construction land area experienced stable growth along with urbanization (0.6% and 2%, respectively).

**Figure 4:**
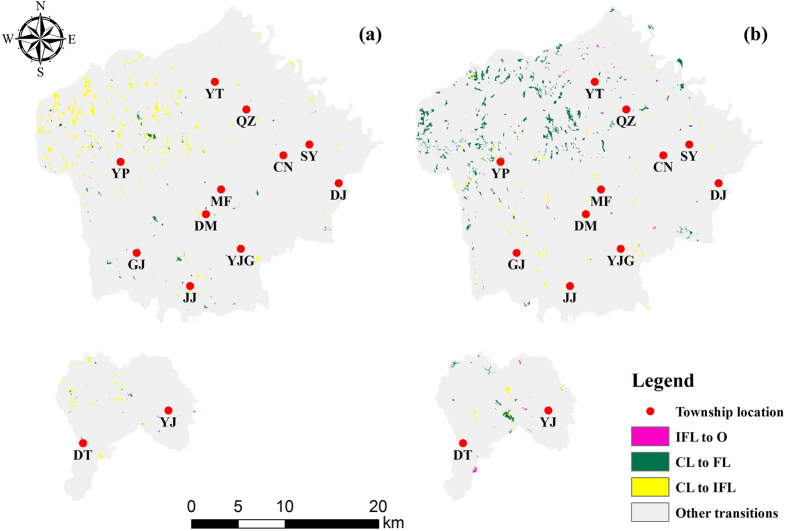
Random land cover transitions from 1992–2000 (a) and 2000–2013 (b). The red points represent each village, and the village names are abbreviated in bold. JJ: Jianjun; DT: Diantou; CN: Changning; YJ: Yijing; GJ: Ganjing; YJG: Yujiagong: DM: Duma; MF: Mafang; SY: Shangyi; DJ: Doujia; QZ: Quzi; YT: Yongtai; YP: Yongping. The maps were generated with ArcGIS 10.2: http://www.esri.com/.

Although many studies have focused on the systematic processes of land cover change^33^,^41^, the study of random changes in land cover has great potential for providing insight into the processes of land cover change in important areas. In the early stages of ecological restoration programmes in Yongping (YP), the growth of forest land area was much slower than that of cultivated land and orchards (Fig. 4a). The ecological restoration programmes were more thoroughly implemented from 2000–2013, as a result, YP, Yongtai (YT) and Quzi (QZ) showed significant transitions from immature forest land and cultivated land to forest land (Fig. 4b).

### Spatial dynamics of changes in land cover

Changes in land cover always have spatially heterogeneous driving forces^42^,^43^. Regions that experience changes in land cover show more rapid changes in some regions than in others^44^. From 1992–2000, forest land presented low spatial dynamics, and the most active regions were the townships of Changning (CN), Duma (DM) and Jianjun (JJ), representing a small area. The most active region was JJ in the county of Yongshou, which is a densely populated area that experienced gradual population growth.

From 2000–2013, the most active regions were mainly distributed in CN, Shangyi (SY), Doujia (DJ), DM, Mafang (MF), Diantou (DT) and Yijing (YJ). All of these areas consisted of cultivated lands and orchards. Forest land also showed low spatial dynamics; for example, YP is a forest zone in Yongshou in which the ecological restoration programmes were primarily implemented, and it presented more active spatial dynamics in this period compared with the previous period. During this period, the regions with high and significant activity expanded to a larger area.

## Discussion

Although the environment has been improved, natural disasters, such as the great flood of 1998^45^ and the spring sandstorm in 2000^46^, have occurred recently in China. These disasters encouraged a policy of continuous ecological restoration and environmental protection programmes in China and caused considerable changes in land cover^47^,^48^. An analysis of regional changes in land cover, structure and spatial characteristics is important and essential for policy-making and ecological management^49^,^50^.

To correctly describe the land cover change and detect the transition mechanism, a high accuracy classification map is needed (Table S2). For example, the high significance of the transition from orchards to construction land is difficult to interpret and is potentially due to classification uncertainties. The classification uncertainties in the studies mainly arise from two sources. One source is the quality of primary TM images and the subsequent pre-processing. This is the main source of classification uncertainty and directly affects the development of classification rules. The second source is the limitations of current classification rules based on object-based methods, including the scale selection of image segmentation, feature selection and classification threshold decision.

This study used an enhanced transition matrix and a modified spatial dynamic degree model to improve the identification and quantification of land cover change. A comparison of the two periods indicated that the study region has experienced a more obvious systematic expansion of forest land, orchards and construction land. The rapid expansion of forest land reflects the success of ecological restoration programmes, and the expansion of orchards and construction land reflect rapid economic development. Severe soil and water losses in this area have placed enormous pressure on social and economic development^51^. The rapid development of the economy has led to rapid urbanization^52^; hence, although the construction land area occupies a very small proportion of the study area, an obvious growth trend was evident. Starting in 2000, a series of ecological restoration programmes were implemented by the Chinese government^53^ and caused significant increases in the conversion of immature forest land and cultivated land to forest land (Figure S1). In addition, an economic development programme caused an increase in the orchard area. The social development and economic growth rate in Yongshou are reflected in the >16-fold increase in GDP from 1992–2000 to 2000–2013^54^, indicating increased urbanization with more residential and construction land.

Many scholars have used the traditional dynamic degree model to monitor the annual average rates of change in land cover^55^,^56^,^57^. Obvious benefits of this model are that its application does not require complex professional skills^58^ and that it can be widely applied to many regions. However, its shortcomings are difficult to overcome: (1) it ignores land cover location and provides little indication of spatial processes and the relative properties of changes in land cover dynamics^36^,^59^, and (2) it only considers one-way transitions and may not detect specific transition patterns. For example, it fails to measure land types that transfer slowly and grow rapidly, such as construction land^60^ (Figure S4).

**Figure 5:**
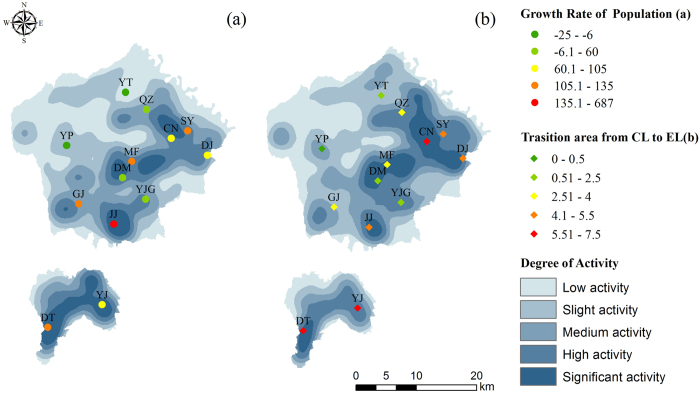
Spatial dynamics of the changes in land cover and relationships with the driving forces from 1992–2000 (a) and 2000–2013 (b). The coloured points represent each village, and the village names are abbreviated in bold. The maps were generated with ArcGIS 10.2: http://www.esri.com/.

Therefore, we considered the spatial location of the process of land cover type changes and proposed a modified spatial analysis model of dynamic changes in land cover based on the traditional model. These results revealed significant associations between the annual population growth rate and the cultivated land area to orchards transition. Forest lands were mainly distributed on higher ground, and the dynamics were minor because of limited human activity. Construction and cultivated land were mainly distributed on plains with soil deposits, and they were more strongly affected by human activity than was forest land, which caused more active dynamics in these areas. Human activities played an important role in the land cover changes. After the reform and opening-up of China, rapid development occurred, and the people’s enthusiasm was mobilized^61^. The main factors that affected the land cover change dynamics from 1992–2000 included agriculture, mining, and urbanization. The government built a series of nature reserves and produced a reasonable plan for urban areas, thus accounting for the more active land cover dynamics in 2000–2013. The land cover dynamics were likely caused by the population flow from the countryside to the town centres from 1992–2000, which is consistent with the population growth rate (Fig. 5a). The spatial pattern of land cover dynamics was consistent with the newly increased area from cultivated land to orchards; therefore, it was likely driven by governmental policies that encouraged the development of orchards (Fig. 5b). In summary, the population growth rate and land cover policies may greatly affect the cultivated land and orchards in this area, and the forest land landscape was more stable during the two studied periods.

We attempted to identify regional changes in land cover that showed systematic and random transitions and to precisely determine the dynamic degrees of land cover change. The results presented here show that the applied method is simple and effective and can identify the relationships between patterns and processes. The method allows the in-depth exploration of driving factors and mechanisms, which can be used to define alternative land covers for further analysis. Future research will focus on in-depth analysis of the underlying driving forces and operating mechanisms to assess the ecological effects of land cover changes on the Loess Plateau. This will help better interpret the mechanism of land cover changes in this study.

## Methods

### Study region

**Figure 6:**
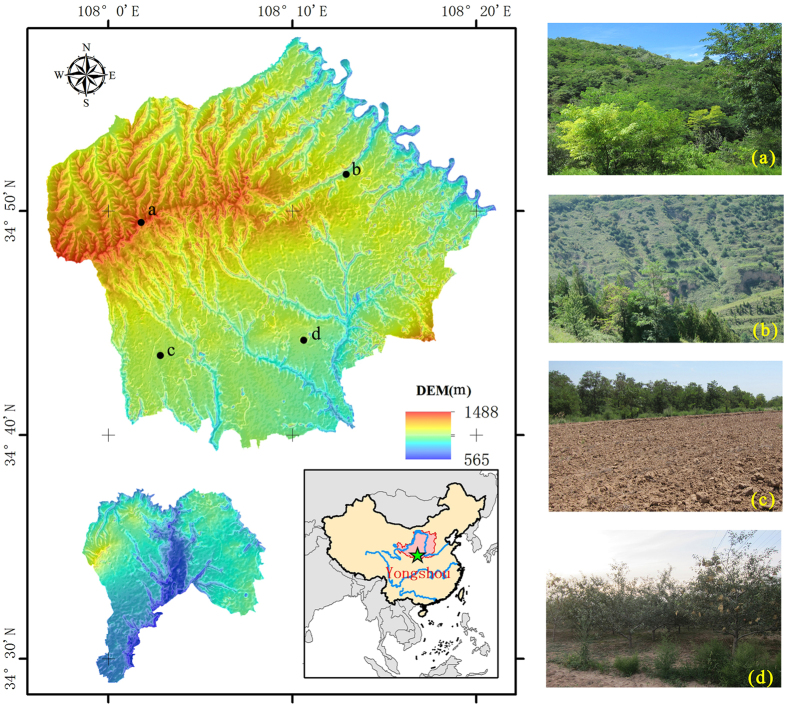
Location of the study area. Photograph of the principal land cover types in Yongshou: (**a**) forest land; (**b**) immature forest land; (**c**) cultivated land; (**d**) orchard. The maps were generated with ArcGIS 10.2: http://www.esri.com/. The right four photos were taken by ZJ.

Yongshou County has a surface area of 885.74 km^2^ and is located at 34°29′02″–34°59′00″N, 107°56′40″–108°20′48″E in the mid-west of Shaanxi Province on the Loess Plateau in China. This area has a warm and semi-humid continental monsoon climate and is characterized by four separate seasons. The summer season is short with dry heat, and the winter season is long and cold. The annual average temperature is 10.8 °C, and the annual rainfall is 578.1–661.3 mm (Fig. 6).

### Data

Three Landsat images from 1992, 2000 and 2013 captured during dry, cloud–free conditions were downloaded from the NASA website (https://www.nasa.gov/) (Table S1). Radiometric and geometric corrections were applied for image enhancement to improve the results of the image classification. The entire area was classified into six land cover types (Table S3) using an object-based classification method with the support of eCognition 8.4^62^,^63^ (Figure S2). Image segmentation was performed to design classification rules and to revise the results of the sample observations. A total of 360 points collected in July 2012 were used to determine the classification results (Figure S3). The overall accuracy of the three classification maps was greater than 90% because of repetitive adjustments prior to the analysis (Tables S2 and S3).

### Land cover transition matrix and assessment

To assess the changes in land cover, we produced two transition matrixes to compare the two periods. A series of methods proposed by Pontius^36^ and Braimoh^59^ were applied in our research. The proportion of a land cover category at time 1 in the transition matrix analysis is determined as follows:


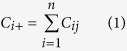


where *C*_*ij*_ (i ≠ j) indicates the proportion of land cover that experienced a transition from class i to class j between time 1 and time 2. The diagonal elements *C*_*jj*_ indicate the proportion of land cover that showed the persistence of class j. Similarly the proportion of the land cover category at time 2 is determined as follows:


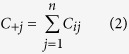


The loss column (Loss) was calculated as the difference between *C*_*i*+_ and the persistence, which indicates a land cover type that experienced a gross loss of class i and the gain row (Gain) was calculated as the difference between *C*_+*j*_ and the persistence, which experienced a gross gain of class j.


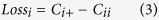



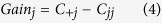


The difference between gains and losses is the net change, which is denoted as *N*_*j*_ = |*C*_+*j*_ − *C*_+*j*_| and represents the most common metric for analysing changes in land cover. However, the net change does not completely reflect the dynamic evolution process of land cover because it fails to consider whether the loss of a category may be replaced with another in the same area at the same time (*N*_*j*_ = 0). This change information is known as a swap. Incorporation of the swap concept avoids underestimating the extent of changes in land cover. Swap is denoted as *S*_*j*_.


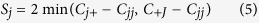


A swap implies a change in the location of a category without a change in the quantity. The swap is twice the gain or loss when the net change is zero.

The total change for each category (*TC*_*j*_) was calculated as either the sum of the net changes and the swap or the sum of the gains and losses:


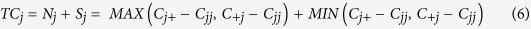


### Detecting the predominant signals of change

The traditional way of identifying the most prominent types of transition is by ranking each conversion between classes after summing up the total area changed during each period. However, this approach fails to consider the presence of the largest categories. To distinguish between systematic and random transitions using a statistical approach, the inter-category transitions must be calculated by summing the total area changed during each period, and the largest categories must be considered. Prominent transitions in quantity may not be a sufficient condition for identifying systematic transitions because even random transitions can cause a large transition area between the largest categories. An in-depth analysis of the transition matrix was used to separate the systematic and random transitions in different periods. This analysis represents a common statistical method that uses the difference between observed and expected values to detect important information. The expected gain and loss values for a random process were calculated by formulas (7) and (8), respectively^33^:


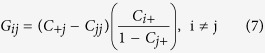



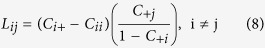


Based on the expected gains (*G*_*ij*_) and expected losses (*L*_*ij*_), we calculated the difference between the observed value and the expected value (*C*_*ij*_ − *G*_*ij*_ or *C*_*ij*_ − *L*_*ij*_) to detect the important information, which was denoted as D_ij_. Deviations equal to or close to zero indicate random inter-category transitions, and large positive or negative deviations indicate systematic transitions between categories. More positive D_ij_ values for a random process for the transition between classes i and j indicate a greater transition area for class i to systematically transition to class j. More negative D_ij_ values for a random process for the transition between classes i and j indicate a greater transition area for class i to avoid systematically transitioning to class j.

D_ij_ can only indicate an inter-category transition tendency rather than the strength of the systematic transition. To solve this problem, the ratio of D_ij_ and the expected value (R_ij_) was used to eliminate the effect of the area proportion of the transition area. More positive R_ij_ values indicate a greater tendency for a systematic transition, whereas more negative R_ij_ values indicate a greater tendency to avoid a systematic transition.

### Dynamics of changes in land cover

To better understand the spatial dynamics of changes in land cover during the two different periods, we modified the dynamic degree model of the changes in land cover proposed by Liu^32^ (changes in land cover dynamic index, LUCDI) at the village level with four steps. In the first step, for each village, we calculate the Increase_i_, Decrease_i_ and Nochange_i_ values of all land cover types in Yongshou to obtain LUCDI (Equation 9). In the second step, the geometric centre of the village is found and the results of step 1 are assigned to the centre points. In the third step, we interpolate the points throughout the entire area using a Kernel Density analysis method (ArcGIS 10.2, http://www.esri.com). The fourth step involves exploring and discussing the spatial relationship between land cover dynamics and possible driving forces, including the rate of population variation and the transition trend caused by policy.


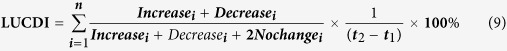


Here, Increase_i_ represents the transitions from j to i where j traverses from 1 to 6 and j is not equal to i, Decrease_i_ represents the transitions from j to i where j traverses from 1 to 6 and j is not equal to i, Nochange_i_ represents the transition from i to i and t_1_ and t_2_ represent different times. LUCDI ranges from 0 to 1. More details regarding the differences between the modified model and the traditional model are presented in Supplementary Information.

## Additional Information

**How to cite this article:** Zhao, J. *et al*. Effects of ecological restoration projects on changes in land cover: A case study on the Loess Plateau in China. *Sci. Rep.* **7**, 44496; doi: 10.1038/srep44496 (2017).

**Publisher's note:** Springer Nature remains neutral with regard to jurisdictional claims in published maps and institutional affiliations.

## Supplementary Material

Supplementary Information

## Figures and Tables

**Table 1:** Area percentage, gains (Gain), losses (Loss), net change (N_j_), swap change (S_j_) and total change (TC_j_) of each land cover type during the two periods.

	FL	IFL	CL	O	CoL	W	C_i+_	Loss	Nj	Sj	TCj
**1992–2000**											
FL	17.85	0.06	0.01	0.00	0.01	0.00	17.93	0.08	3.30	0.16	3.46
IFL	3.11	30.06	0.37	0.01	0.09	0.01	33.66	3.60	2.20	2.78	4.98
CL	0.23	1.29	34.62	6.08	0.16	0.00	42.39	7.77	7.33	0.88	8.21
O	0.00	0.00	0.01	3.38	0.30	0.00	3.69	0.31	5.82	0.62	6.45
CoL	0.00	0.03	0.03	0.03	1.84	0.00	1.94	0.10	0.47	0.20	0.67
W	0.03	0.01	0.01	0.00	0.00	0.33	0.38	0.06	0.04	0.02	0.06
C_+j_	21.23	31.46	35.06	9.51	2.41	0.34	100.00				
Gain	3.38	1.39	0.44	6.13	0.56	0.01					
**2000–2013**											
FL	20.80	0.01	0.13	0.02	0.20	0.03	21.23	0.39	18.95	0.78	19.73
IFL	17.71	12.59	0.36	0.07	0.71	0.01	31.46	18.84	18.41	0.86	19.27
CL	1.58	0.42	23.25	8.95	0.85	0.01	35.06	11.74	11.23	1.02	12.25
O	0.00	0.00	0.02	9.05	0.43	0.00	9.51	0.47	8.57	0.94	9.51
CoL	0.00	0.00	0.01	0.00	2.41	0.00	2.41	0.03	2.18	0.06	2.24
W	0.04	0.01	0.00	0.00	0.00	0.29	0.34	0.05	0.00	0.10	0.10
C_+j_	40.17	13.02	23.77	18.10	4.61	0.34	100.00				
Gain	19.34	0.43	0.51	9.04	2.21	0.05					

The diagonal elements represent the persistence under random change.

**Table 2:** Percentages of changes in land cover in terms of gains and losses for each period.

		FL		IFL		CL		O		CoL		W	
1992–2000		gain	loss	gain	loss	gain	loss	gain	loss	gain	loss	gain	loss
FL	D_ij_	0.00	0.00	−0.28	0.03	−0.13	−0.03	−1.14	−0.01	−0.09	0.01	0.00	0.00
	R_ij_	0.00	0.00	−0.82	1.00	−0.93	−0.75	−1.00	−1.00	−0.90	0.00	0.00	0.00
IFL	D_ij_	1.72	1.99	0.00	0.00	0.11	−1.47	−2.13	−0.49	−0.10	−0.04	0.01	−0.01
	R_ij_	1.24	1.78	0.00	0.00	0.42	−0.80	−1.00	−0.98	−0.53	−0.31	0.00	−0.50
CL	D_ij_	−1.52	−2.31	0.40	−2.47	0.00	0.00	3.38	4.94	−0.08	−0.13	0.00	−0.04
	R_ij_	−0.87	−0.91	0.45	−0.66	0.00	0.00	1.25	4.33	−0.33	−0.45	0.00	−1.00
O	D_ij_	−0.15	−0.07	−0.08	−0.11	−0.02	−0.11	0.00	0.00	0.28	0.29	0.00	0.00
	R_ij_	−1.00	−1.00	−1.00	−1.00	−0.67	−0.92	0.00	0.00	14.00	29.00	0.00	0.00
CoL	D_ij_	−0.08	−0.02	−0.01	0.00	0.02	−0.01	−0.09	0.02	0.00	0.00	0.00	0.00
	R_ij_	−1.00	−1.00	−0.25	0.00	2.00	−0.25	−0.75	2.00	0.00	0.00	0.00	0.00
W	D_ij_	0.01	0.02	0.00	−0.01	0.01	−0.01	−0.02	−0.01	0.00	0.00	0.00	0.00
	R_ij_	0.50	2.00	0.00	−0.50	0.00	−0.50	−1.00	−1.00	0.00	0.00	0.00	0.00
**2000–2013**													
FL	D_ij_	0.00	0.00	−0.12	−0.07	−0.04	−0.02	−2.10	−0.10	−0.28	0.17	0.02	0.03
	R_ij_	0.00	0.00	−0.92	−0.88	−0.24	−0.13	−0.99	−0.83	−0.58	5.67	2.00	0.00
IFL	D_ij_	9.99	9.01	0.00	0.00	0.11	−4.79	−3.07	−3.85	0.00	−0.29	−0.01	−0.06
	R_ij_	1.29	1.04	0.00	0.00	0.44	−0.93	−0.98	−0.98	0.00	−0.29	−0.50	−0.86
CL	D_ij_	−7.03	−4.61	0.20	−1.59	0.00	0.00	5.45	6.16	0.06	0.14	−0.01	−0.04
	R_ij_	−0.82	−0.74	0.91	−0.79	0.00	0.00	1.56	2.21	0.08	0.20	−0.50	−0.80
O	D_ij_	−2.33	−0.23	−0.06	−0.07	−0.05	−0.12	0.00	0.00	0.21	0.40	0.00	0.00
	R_ij_	−1.00	−1.00	−1.00	−1.00	−0.71	−0.86	0.00	0.00	0.95	13.33	0.00	0.00
CoL	D_ij_	−0.59	−0.01	−0.02	0.00	−0.01	0.00	−0.24	−0.01	0.00	0.00	0.00	0.00
	R_ij_	−1.00	−1.00	−1.00	0.00	−0.50	0.00	−1.00	−1.00	0.00	0.00	0.00	0.00
W	D_ij_	−0.04	0.02	0.01	0.00	0.00	−0.01	−0.03	−0.01	−0.01	0.00	0.00	0.00
	R_ij_	−0.50	1.00	0.00	0.00	0.00	1.00	−1.00	−1.00	−1.00	0.00	0.00	0.00

The difference between the observed and the expected value (D_ij_) is shown along with the difference between the observed and expected value, relative to the expected value (R_ij_) under a random change process from the perspective of gains (%) and losses (%).

## References

[b1] Foley J. A. . Global consequences of land use. Science 309, 570–574 (2005). 16040698 10.1126/science.1111772

[b2] Turner B. L. 2nd, Lambin E. F. & Reenberg A. The emergence of land change science for global environmental change and sustainability. Proc Natl Acad Sci USA 104, 20666–20671, doi: 10.1073/pnas.0704119104 (2007). 18093934 PMC2409212

[b3] Barnes A. D. . Consequences of tropical land use for multitrophic biodiversity and ecosystem functioning. Nat Commun 5, 5351, doi: 10.1038/ncomms6351 (2014). 25350947 PMC4220457

[b4] Nosetto M. D., Jobbágy E. G., Brizuela A. B. & Jackson R. B. The hydrologic consequences of land cover change in central Argentina. Agriculture, Ecosystems & Environment 154, 2–11, doi: 10.1016/j.agee.2011.01.008 (2012).

[b5] Poulter B. . Sensitivity of global terrestrial carbon cycle dynamics to variability in satellite-observed burned area. Global Biogeochem Cy 29, 207–222 (2015).

[b6] Kirschbaum M. U., Saggar S., Tate K. R., Thakur K. P. & Giltrap D. L. Quantifying the climate-change consequences of shifting land use between forest and agriculture. The Science of the total environment 465, 314–324, doi: 10.1016/j.scitotenv.2013.01.026 (2013). 23419358

[b7] Defries R. & Bounoua L. Consequences of land use change for ecosystem services: A future unlike the past. Geojournal 61, págs. 345–351 (2004).

[b8] Turner B. L. I. . Land-use and land-cover change. Science/Research plan. Global Change Report 43, 669–679 (1995).

[b9] Secretariat I., Scholes M., Doherty S. & Young B. Science Plan and Implementation Strategy. Environmental Policy Collection 20, 1262–1268 (2006).

[b10] Zhou P. . Changes in land use and agricultural production structure before and after the implementation of grain for green program in Western China - taking two typical counties as examples. J Mt Sci 11, 526–534, doi: 10.1007/s11629-013-2369-2 (2014).

[b11] FANG J.-Q. & XIE Z. Deforestation in preindustrial China: the loess plateau region as an example. (Elsevier, 1994).

[b12] Zheng F., He X., Gao X., Zhang C.-e. & Tang K. Effects of erosion patterns on nutrient loss following deforestation on the Loess Plateau of China. Agriculture, Ecosystems & Environment 108, 85–97, doi: 10.1016/j.agee.2004.12.009 (2005).

[b13] Zhao G., Liu J., Kuang W., Ouyang Z. & Xie Z. Disturbance impacts of land use change on biodiversity conservation priority areas across China: 1990–2010. J Geogr Sci 25, 515–529, doi: 10.1007/s11442-015-1184-9 (2015).

[b14] Zhao G., Mu X., Wen Z., Wang F. & Gao P. Soil Erosion, Conservation, and Eco-Environment Changes in the Loess Plateau of China. Land Degrad Dev, n/a–n/a, doi: 10.1002/ldr.2246 (2013).

[b15] Wang X., Shen J. & Zhang W. Emergy evaluation of agricultural sustainability of Northwest China before and after the grain-for-green policy. Energ Policy 67, 508–516, doi: 10.1016/j.enpol.2013.12.060 (2014).

[b16] Xu Z. G., Bennett M. T., Tao R. & Xu J. T. China’s Sloping Land Conversion Programme four years on: current situation and pending issues. Int For Rev 6, 317–326, doi: 10.1505/ifor.6.3.317.59976 (2004).

[b17] Zhou H. Y. People’s Daily editorial office. http://www.people.com.cn/GB/huanbao/55/20011126/613279.html (2001).

[b18] Karan S. K. & Samadder S. R. Accuracy of land use change detection using support vector machine and maximum likelihood techniques for open-cast coal mining areas. Environ Monit Assess 188, 486, doi: 10.1007/s10661-016-5494-x (2016). 27461425

[b19] Zhao J. S., Yuan L. & Zhang M. A study of the system dynamics coupling model of the driving factors for multi-scale land use change. Environmental Earth Sciences 75, doi: 10.1007/s12665-015-5165-1 (2016).

[b20] Chen N., Ma T. & Zhang X. Responses of soil erosion processes to land cover changes in the Loess Plateau of China: A case study on the Beiluo River basin. Catena 136, 118–127, doi: 10.1016/j.catena.2015.02.022 (2016).

[b21] Zhou D., Zhao S. & Zhu C. The Grain for Green Project induced land cover change in the Loess Plateau: A case study with Ansai County, Shanxi Province, China. Ecol Indic 23, 88–94, doi: 10.1016/j.ecolind.2012.03.021 (2012).

[b22] Wang S., Zhang Z., Sun G., McNulty S. G. & Zhang M. Detecting water yield variability due to the small proportional land use and land cover changes in a watershed on the Loess Plateau, China. Hydrol Process 23, 3083–3092, doi: 10.1002/hyp.7420 (2009).

[b23] Zhou J. . Effects of precipitation and restoration vegetation on soil erosion in a semi-arid environment in the Loess Plateau, China. Catena 137, 1–11, doi: 10.1016/j.catena.2015.08.015 (2016).

[b24] Ouyang Z. . Improvements in ecosystem services from investments in natural capital. Science 352, 1455–1459, doi: 10.1126/science.aaf2295 (2016). 27313045

[b25] Feng X. . Revegetation in China’s Loess Plateau is approaching sustainable water resource limits. Nature Climate Change 6, 1019–1022, doi: 10.1038/nclimate3092 (2016).

[b26] Wang S. . Reduced sediment transport in the Yellow River due to anthropogenic changes. Nat Geosci 9, 38–41, doi: 10.1038/ngeo2602 (2015).

[b27] Deng L., Shangguan Z. P. & Sweeney S. “Grain for Green” driven land use change and carbon sequestration on the Loess Plateau, China. Sci Rep 4, 7039, doi: 10.1038/srep07039 (2014). 25391219 PMC4229669

[b28] Shi S. & Han P. Estimating the soil carbon sequestration potential of China’s Grain for Green Project. Global Biogeochem Cy 28, 2211–2215 (2014).

[b29] Peng S. S. . Afforestation in China cools local land surface temperature. Proc Natl Acad Sci USA 111, 2915–2919, doi: 10.1073/pnas.1315126111 (2014). 24516135 PMC3939881

[b30] Sun W. . Spatiotemporal vegetation cover variations associated with climate change and ecological restoration in the Loess Plateau. Agricultural and Forest Meteorology 209–210, 87–99, doi: 10.1016/j.agrformet.2015.05.002 (2015).

[b31] Vulpiani A. Andrey Andreyevich Markov: a furious mathematician and his chains. Lettera Matematica 3, 205–211, doi: 10.1007/s40329-015-0099-8 (2015).

[b32] Liu J. & Buheaosier. Study on spatial-temporal feature of modern land-use change in China using remote sensing techiques. China Academic Journal 20, 230–239 (2000).

[b33] Teferi E., Bewket W., Uhlenbrook S. & Wenninger J. Understanding recent land use and land cover dynamics in the source region of the Upper Blue Nile, Ethiopia: Spatially explicit statistical modeling of systematic transitions. Agriculture, ecosystems & environment 165, 98–117 (2013).

[b34] Mertens B. & Lambin E. F. Land-Cover-Change Trajectories in Southern Cameroon. Ann Assoc Am Geogr 90, 467–494, doi: 10.1111/0004-5608.00205 (2000).

[b35] Ouedraogo I., Savadogo P., Tigabu M., Dayamba S. D. & Odén P. C. Systematic and random transitions of land-cover types in Burkina Faso, West Africa. International Journal of Remote Sensing 32, 5229–5245, doi: 10.1080/01431161.2010.495095 (2011).

[b36] Pontius R. G., Shusas E. & McEachern M. Detecting important categorical land changes while accounting for persistence. Agriculture, Ecosystems & Environment 101, 251–268 (2004).

[b37] Zhang J. & Ren Z. Spatiotemporal pattern and terrain gradient effect of land use change in Qinling-Bashan mountains. Transactions of the Chinese Society of Agricultural Engineering 32, 250–257, doi: 10.11975/j.issn.1002-6819.2016.14.033 (2016).

[b38] Yang C., Shen W. & Wang T. Spatial-temporal characteristics of cultivated land in Tibet in recent 30 years. Transaction of the Chinese Society of Agricultural Engineering 31, 264–271 (2015).

[b39] Chang C., Zhao G., Wang L., Zhu X. & Li T. Land use spatiotemporal changes and its driving forces analysis in vulnerable ecological region of Yellow River Estuary. Transactions of the Chinese Society of Agricultural Engineering 28, 226–234, doi: 10.3969/j.issn.1002-6819.2012.24.031 (2012).

[b40] Yi L. . Spatial-temporal change of major reserve resources of cultivated land in China in recent 30 years. Transactions of the Chinese Society of Agricultural Engineering 29, 1–12, doi: 10.3969/j.issn.1002-6819.2013.06.001 (2013).

[b41] van Vliet J., de Groot H. L. F., Rietveld P. & Verburg P. H. Manifestations and underlying drivers of agricultural land use change in Europe. Landscape Urban Plan 133, 24–36, doi: 10.1016/j.landurbplan.2014.09.001 (2015).

[b42] Celio E., Koellner T. & Grêt-Regamey A. Modeling land use decisions with Bayesian networks: Spatially explicit analysis of driving forces on land use change. Environmental Modelling & Software 52, 222–233, doi: 10.1016/j.envsoft.2013.10.014 (2014).

[b43] Gao P., Niu X., Wang B. & Zheng Y. Land use changes and its driving forces in hilly ecological restoration area based on gis and RS of northern China. Sci Rep 5, 11038, doi: 10.1038/srep11038 (2015). 26047160 PMC4457013

[b44] Hlásny T., Trombik J., Dobor L., Barcza Z. & Barka I. Future climate of the Carpathians: climate change hot-spots and implications for ecosystems. Reg Environ Change 16, 1495–1506, doi: 10.1007/s10113-015-0890-2 (2015).

[b45] Zong Y. & Chen X. The 1998 flood on the Yangtze, China. Nat Hazards 22, 165–184, doi: 10.1023/a:1008119805106 (2000).

[b46] Zhou X., Xu X., Yan P., Weng Y. & Wang J. Dynamic characteristics of spring sandstorms in 2000. Science in China Series D: Earth Sciences 45, 921–930, doi: 10.1360/02yd9091 (2002).

[b47] Huang C. . Satellite data regarding the eutrophication response to human activities in the plateau lake Dianchi in China from 1974 to 2009. The Science of the total environment 485–486, 1–11, doi: 10.1016/j.scitotenv.2014.03.031 (2014). 24698830

[b48] Baker S. & Eckerberg K. Ecological restoration success: a policy analysis understanding. Restor Ecol 24, 284–290, doi: 10.1111/rec.12339 (2016).

[b49] Liu Y., Fang F. & Li Y. Key issues of land use in China and implications for policy making. Land Use Policy 40, 6–12, doi: 10.1016/j.landusepol.2013.03.013 (2014).

[b50] Xiao R., Wang G., Zhang Q. & Zhang Z. Multi-scale analysis of relationship between landscape pattern and urban river water quality in different seasons. Sci Rep 6, 25250, doi: 10.1038/srep25250 (2016). 27147104 PMC4857082

[b51] Ren Z., Zhu L., Wang B. & Cheng S. Soil hydraulic conductivity as affected by vegetation restoration age on the Loess Plateau, China. Journal of Arid Land 8, 546–555, doi: 10.1007/s40333-016-0010-2 (2016).

[b52] Shi M. J. & Chen K. Land degradation, government subsidy, and smallholders’ conservation decision: the case of the loess plateau in China. J Zhejiang Univ Sci 5, 1533–1542, doi: 10.1631/jzus.2004.1533 (2004). 15547961

[b53] Dang X., Liu G., Xue S. & Li P. An ecological footprint and emergy based assessment of an ecological restoration program in the Loess Hilly Region of China. Ecol Eng 61, 258–267, doi: 10.1016/j.ecoleng.2013.09.018 (2013).

[b54] Zhao Y. H. Yongshou government website, http://www.yongshou.gov.cn/html/gov/4/ghjhjc/66085/66085.html (2015).

[b55] Muriithi F. K. Land use and land cover (LULC) changes in semi-arid sub-watersheds of Laikipia and Athi River basins, Kenya, as influenced by expanding intensive commercial horticulture. Remote Sensing Applications: Society and Environment 3, 73–88, doi: 10.1016/j.rsase.2016.01.002 (2016).

[b56] Hassan M. M. & Nazem M. N. I. Examination of land use/land cover changes, urban growth dynamics, and environmental sustainability in Chittagong city, Bangladesh. Environment, Development and Sustainability 18, 697–716, doi: 10.1007/s10668-015-9672-8 (2015).

[b57] Gilani H. . Decadal land cover change dynamics in Bhutan. J Environ Manage 148, 91–100, doi: 10.1016/j.jenvman.2014.02.014 (2015). 24680540

[b58] Alo C. A. & Pontius R. G. Jr. Identifying systematic land-cover transitions using remote sensing and GIS: the fate of forests inside and outside protected areas of Southwestern Ghana. Environment and planning. B, Planning & design 35, 280 (2008).

[b59] Braimoh A. K. Random and systematic land-cover transitions in northern Ghana. Agriculture, ecosystems & environment 113, 254–263 (2006).

[b60] Liu S. & He S. A spatial analysis model for measuring the rate of land use change. Journal of natural resources 17, 533–540 (2002).

[b61] Qian C. Impact of land use/land cover change on changes in surface solar radiation in eastern China since the reform and opening up. Theor Appl Climatol 123, 131–139, doi: 10.1007/s00704-014-1334-5 (2014).

[b62] Wu B., Xiong Z.-g., Chen Y.-z. & Zhao Y.-d. Classification of quickbird image with maximal mutual information feature selection and support vector machine. Procedia Earth and Planetary Science 1, 1165–1172, doi: 10.1016/j.proeps.2009.09.179 (2009).

[b63] Rasuly A., Naghdifar R. & Rasoli M. Monitoring of Caspian Sea Coastline Changes Using Object-Oriented Techniques. Procedia Environmental Sciences 2, 416–426, doi: 10.1016/j.proenv.2010.10.046 (2010).

