# Supplementary material for: Effects of ecological restoration projects on changes in land cover: A case study on the Loess Plateau in China
Source: Sci Rep. 2017 Mar 21;7:44496. doi: 10.1038/srep44496 (PMC5359563; doi:10.1038/srep44496)
Supplement: Supplementary Information [file srep44496-s1.doc]

**SUPPLEMENTARY INFORMATION**

**Effects of ecological restoration projects on changes in land cover: A case study on the Loess Plateau in China**

Jun Zhao^1,2,3^, Yanzheng Yang^4^, Qingxia Zhao^1,2,3^, Zhong Zhao^1,2,3^*

1. College of Forestry, Northwest A&F University, Yangling, Shaanxi 712100, P. R. China

2. Key Comprehensive Laboratory of Forestry, Shaanxi Province, P. R. China

3. Key Laboratory of Silviculture on the Loess Plateau, State Forestry Administration, Shaanxi Province, P. R. China

4. Ministry of Education Key Laboratory for Earth System Modeling, Department of Earth System Science, Tsinghua University

Correspondence and requests for materials should be addressed to Z.Z. (zhaozh@nwafu.edu.cn)

**Details on the ecological programmes implemented in Yongshou.** Three ecological programmes were implemented in Yongshou during the examined study periods: the Natural Forest Protection Project (NFPP), the Grain for Green Project (GFGP) and the “Three North” Shelterbelt Development Program (TNSDP). The TNSDP was implemented in 1978 and was the sole ecological programme in effect before 2001 (Figure S1). From 2007-2013, the TNSDP was continuously implemented by the local government. The NFPP was primarily implemented from 2001-2005. The GFGP was implemented in 2001 and accounted for a high proportion of the ecological programmes (Figure S1).

**Object-based classification method and post-processing**. Object-based classification is widely employed in land surveying and monitoring and has generated many satisfactory results^1,2^. However, this method has rarely been applied in land cover classification on the Loess Plateau with Landsat images. The object-based technique uses groups of pixels or “objects” instead of individual pixels as the unit of classification, and it assigns each object in an image to a class^3^. Two basic methods can be employed for object-based classification: classification by rules and classification by training samples. Classification by rules was adopted in this study. There are two main steps involved in classification by rules. One entails image segmentation, and the other involves determining the classification threshold. In image segmentation, each pixel as initially considered as an individual object, which is merging with its neighbour object if they have similar characteristics, thereby producing a new, larger object. This progress is repeated until the size of the objects meets the user-specified threshold^3^. We set the segmentation scale at 8 in our study. We first divided all of the objects into forest land and non-forest land based on the Normalized Difference Vegetation Index (NDVI), and we then classified the non-forest land into water and non-water categories using Normalized Difference Water Index (NDWI). We classified construction land and cultivated land based on the Enhanced Index based Built-up Index (EIBI) and the Modified Normalized Difference Water Index (MNDWI). Forest land was classified into orchard and non-orchard with indices of mean slope, and then non-orchard was further classified into immature forest land and forest land with the indices of slope and NDVI (Figure S2).

We first used object-based methods to produce a classification map and then compared it with national forest survey data for this area. We immediately corrected the shape file if any errors occurred. We also compared the data with several known points and high-resolution images from Google Earth to improve the classification accuracy. We ceased classification after good performance was obtained.

**Evaluation of the classification results.** We first used object-based methods to produce a classification map of the study area (Figure S2) and then edited the shape file after comparing it with the survey data to improve the classification accuracy. A total of 408 points were collected; however, 360 of them were unchanged over past 20 years, which was validated by consulting local people or from worker record of the local forest bureau. The final classifications were evaluated by calculating overall accuracy (Eq. S1) and the kappa coefficient (Eq. S2)^5^ :


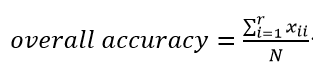
 (S1)


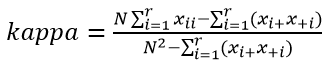
 (S2)

where N is the number of samples used to evaluate the classification results, r is the number of classes, x_i+_ is the total of the i^th^ row in the confusion matrix, x_+i_ is the total of the i^th^ column, and x_ii_ is the value located in the i^th^ row and i^th^ column of the confusion matrix.

To correctly describe the land cover change and detect the transition mechanism, a high accuracy classification map is needed. For example, the high significance of the transition from orchards to construction land is difficult to interpret and is potentially due to classification uncertainties. The classification uncertainties in the studies mainly arise from two sources. One source is the quality of primary TM images and the subsequent pre-processing. This is the main source of classification uncertainty and directly affects the development of classification rules. The second source is the limitations of current classification rules based on object-based methods, including the scale selection of image segmentation, feature selection and classification threshold decision.

**Differences between the modified and traditional dynamic degree model.** The traditional model proposed by Liu^4^ can be used to measure and compare the activity of land cover change and to rapidly determine the intensity of this change at different spatial scales. The formula is as follows:


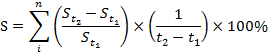


Here, S_t1_ and S_t2_ represent the area at t_1_ and t_2_, respectively; t_1_ and t_2_ represent different times.

The traditional dynamic degree model is unable to detect areas that have undergone changes with no change in area (Figure S4). Three cases are described to illustrate the differences between the modified model and the traditional dynamic degree model. In case 1, the results of the traditional and modified models both equal 0 (Figure S4a); in case 2, the result of the traditional model is 0, but that of the modified model is 0.5 (Figure S4b); and in case 3, the result of traditional model is 0, but that of the modified model is 1 (Figure S4c).

Table S1 Description of the satellite images used. NFPP, Natural Forest Protection Project; GFGP, Grain for Green Project; TNSDP, “Three North” Shelterbelt Development Program.

|  | 1992 | 2000 | 2013 |
| --- | --- | --- | --- |
| Satellite (sensor) | Landsat5 TM | Landsat7 ETM+ | Landsat8 OLI |
| Path/Row | 127/036 | 127/036 | 127/036 |
| Acquisition date | 07/17/1992 | 06/29/2000 | 06/25/2013 |
| Pixel spacing (m) | 30 | 30 | 30 |
| Sun Elevation | 58.79 | 66.01 | 68.34 |
| Sun Azimuth | 107.02 | 111.88 | 114.99 |
| Cloud cover (%) | 2.00 | 0.47 | 0.46 |

Table S2 Classification accuracy in the three periods.

| Year | Overall accuracy (%) | Kappa coefficient |
| --- | --- | --- |
| 1992 | 90.28 | 0.76 |
| 2000 | 91.94 | 0.78 |
| 2013 | 88.61 | 0.72 |

Table S3 Description of land cover classes

| Land cover classes | Description | Code |
| --- | --- | --- |
| Forest land | Areas composed of forests with a canopy density 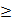0.2 | FL |
| Immature forest land | Areas with recently planted forest lacking a closed canopy | IFL |
| Orchards | Gardens of planted fruit trees | O |
| Cultivated land | Areas covered with annual crops, with periods of harvest and bare soil | CL |
| Construction land | Areas with urban or rural residential buildings, public facilities, mines, roads or other construction land | CoL |
| Water | Areas with water or water facilities | W |

Table S4 Confusion matrix for the results of an object-based classification in 2013. FL, forest land; IFL, immature forest land; CL, cultivated land; O, orchards; CoL, construction land; W, water.

| Types | FL | IFL | CL | O | CoL | W | Total |
| --- | --- | --- | --- | --- | --- | --- | --- |
| FL | 240 | 16 | 5 | 2 | 1 | 0 | 264 |
| IFL | 2 | 32 | 2 | 1 | 1 | 0 | 38 |
| CL | 3 | 1 | 14 | 0 | 0 | 0 | 18 |
| O | 2 | 1 | 3 | 25 | 1 | 0 | 32 |
| CoL | 0 | 0 | 0 | 0 | 5 | 0 | 5 |
| W | 0 | 0 | 0 | 0 | 0 | 3 | 3 |
| Total | 247 | 50 | 24 | 28 | 8 | 3 | 360 |

Table S5 Area statistics for each land cover type in 1992, 2000 and 2013 (km^2^). FL, forest land; IFL, immature forest land; CL, cultivated land; O, orchards; CoL, construction land; W, water.

|  | SF | IFL | CL | O | CoL | W |
| --- | --- | --- | --- | --- | --- | --- |
| 1992 | 173197.44 | 231135.12 | 331333.74 | 52899.48 | 10661.22 | 3928.5 |
| 2000 | 176678.82 | 203257.35 | 325428.03 | 80225.64 | 13978.17 | 3587.49 |
| 2013 | 321807.33 | 92063.79 | 189765.99 | 165329.91 | 28192.05 | 3101.49 |

**Supplementary Figure Captions**

Figure S1 Proportions of the different ecological restoration programmes implemented in the study area.

Figure S2 Flow chart of the steps for identifying land cover types from Landsat TM images.

Figure S3 Locations of the validation points. The maps were generated with ArcGIS 10.2: <http://www.esri.com/>.

Figure S4 Three typical cases of land cover change. (a) no change; (b) spatial shift with overlap and no change in area (decrease equals increase); (c) spatial shift without overlap and no change in area (decrease equals increase).


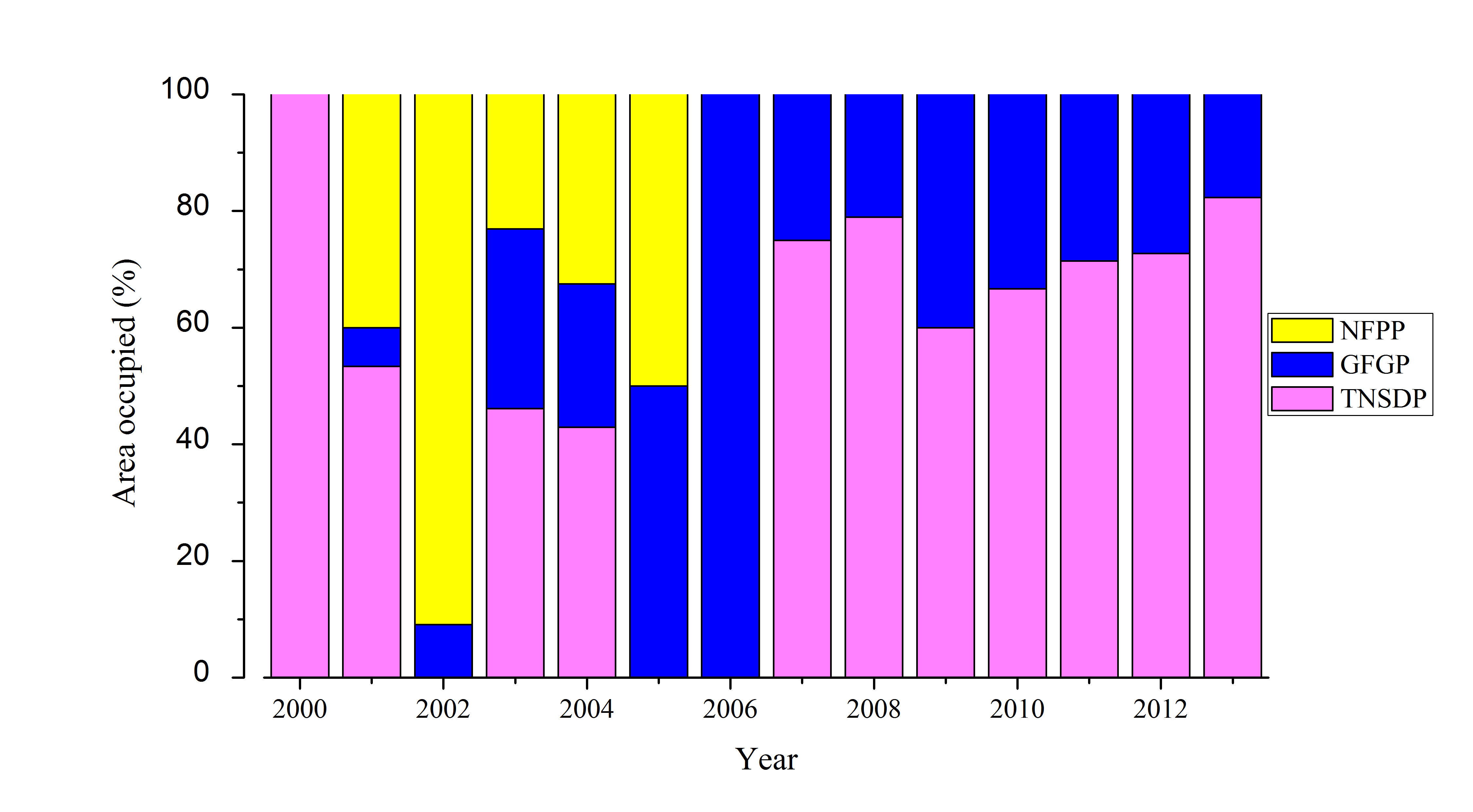


Figure S1


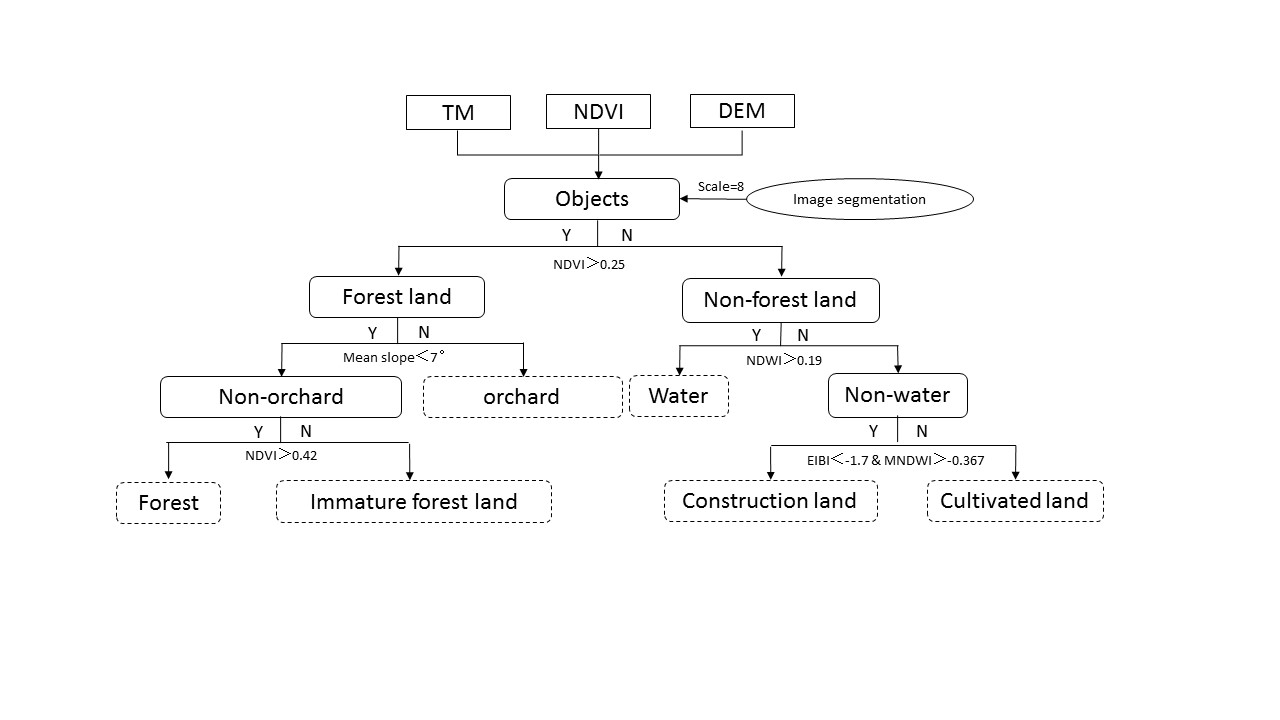


Figure S2


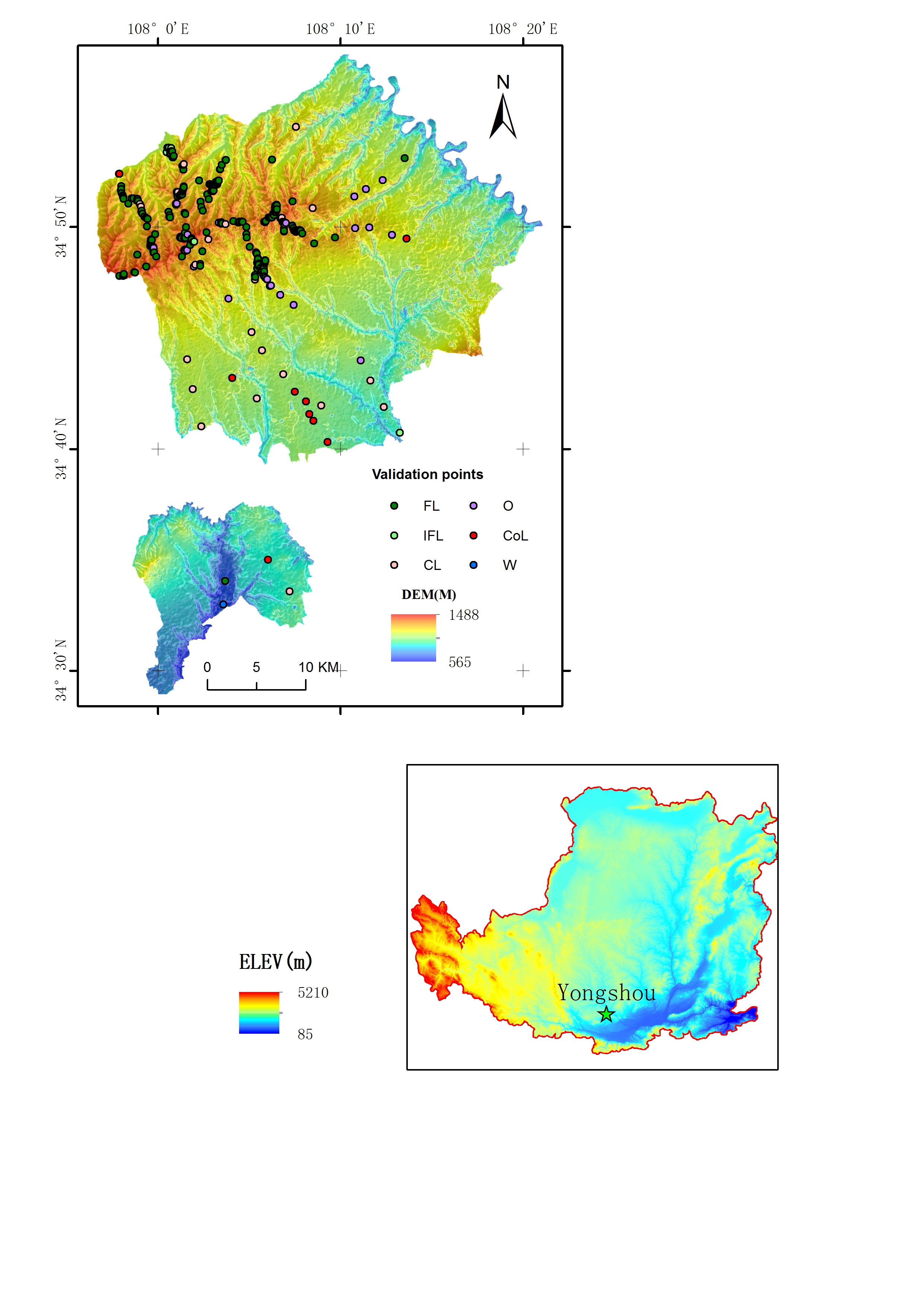


Figure S3

**
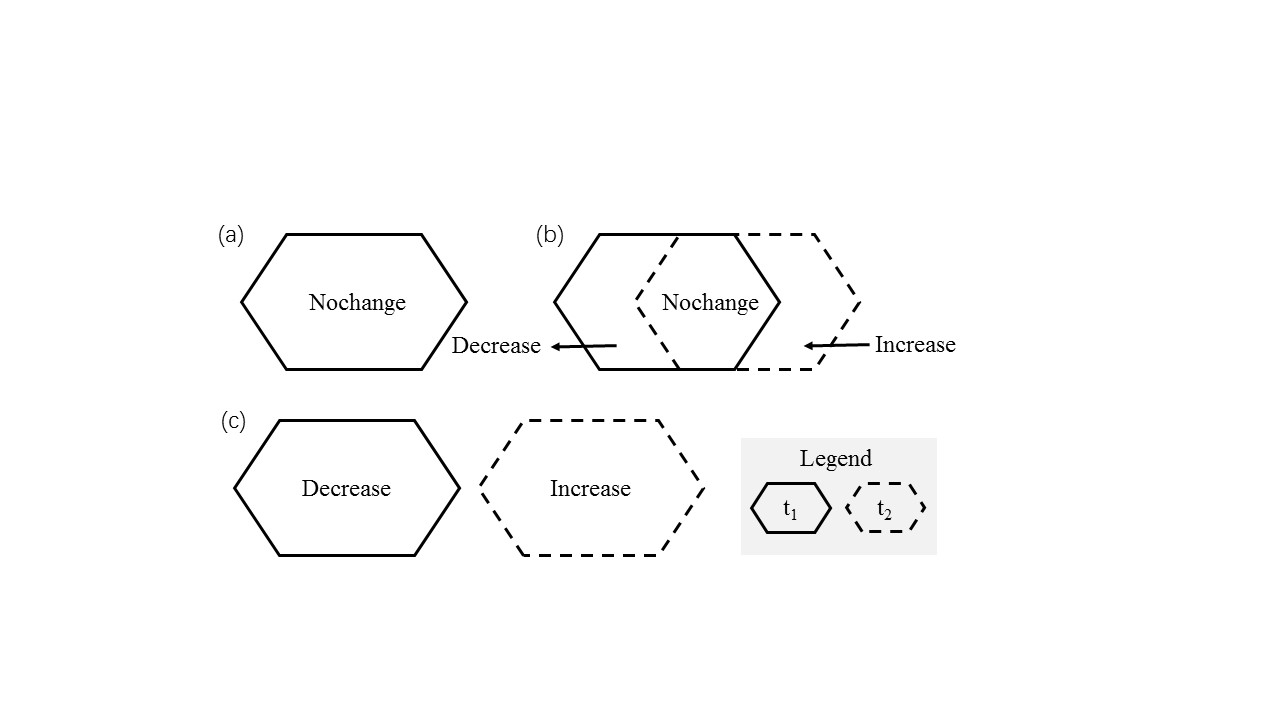
**

Figure S4

**References** (Supplementary Information)

1 van Lier, O. R., Fournier, R. A., Bradley, R. L. & Thiffault, N. A multi-resolution satellite imagery approach for large area mapping of ericaceous shrubs in Northern Quebec, Canada. *International Journal of Applied Earth Observation and Geoinformation* **11**, 334-343, doi:10.1016/j.jag.2009.05.003 (2009).

2 Vancoillie, F., Verbeke, L. & Dewulf, R. Feature selection by genetic algorithms in object-based classification of IKONOS imagery for forest mapping in Flanders, Belgium. *Remote Sensing of Environment* **110**, 476-487, doi:10.1016/j.rse.2007.03.020 (2007).

3 Wulder, M. A., Chubey, M. S. & Franklin, S. E. Object-based Analysis of Ikonos-2 Imagery for Extraction of Forest Inventory Parameters. *Photogrammetric Engineering & Remote Sensing* **72**, págs. 383-394 (2006).

4 Jiyuan, L. & Buheaosier. Study on spatial-temporal feature of modern land-use change in China using remote sensing techiques. *China Academic Journal* **20**, 230-239 (2000).
